# Novel Perspectives towards RNA-Based Nano-Theranostic Approaches for Cancer Management

**DOI:** 10.3390/nano11123330

**Published:** 2021-12-08

**Authors:** Rabia Arshad, Iqra Fatima, Saman Sargazi, Abbas Rahdar, Milad Karamzadeh-Jahromi, Sadanand Pandey, Ana M. Díez-Pascual, Muhammad Bilal

**Affiliations:** 1 Faculty of Pharmacy, University of Lahore, Lahore 45320, Pakistan; rabia.arshad@pharm.uol.edu.pk; 2 Department of Pharmacy, Quaid-i-Azam University, Islamabad 45320, Pakistan; iqraf332@gmail.com; 3 Cellular and Molecular Research Center, Research Institute of Cellular and Molecular Sciences in Infectious Diseases, Zahedan University of Medical Sciences, Zahedan 98167-43463, Iran; sgz.biomed@gmail.com; 4 Department of Physics, University of Zabol, Zabol 98613-35856, Iran; 5 Department of Physics, University of Kashan, Kashan 87317-51167, Iran; milad71karamzade@yahoo.com; 6 Department of Chemistry, College of Natural Science, Yeungnam University, 280 Daehak-Ro, Gyeongsan 38541, Korea; sadanand.au@gmail.com; 7 Universidad de Alcalá, Facultad de Ciencias, Departamento de Química Analítica, Química Física e Ingeniería Química, Ctra. Madrid-Barcelona, Km. 33.6, 28805 Alcalá de Henares, Madrid, Spain; 8 School of Life Science and Food Engineering, Huaiyin Institute of Technology, Huai’an 223003, China; bilaluaf@hotmail.com

**Keywords:** RNA nanotechnology, cancer, theranostic, nano-biosensor

## Abstract

In the fight against cancer, early diagnosis is critical for effective treatment. Traditional cancer diagnostic technologies, on the other hand, have limitations that make early detection difficult. Therefore, multi-functionalized nanoparticles (NPs) and nano-biosensors have revolutionized the era of cancer diagnosis and treatment for targeted action via attaching specified and biocompatible ligands to target the tissues, which are highly over-expressed in certain types of cancers. Advancements in multi-functionalized NPs can be achieved via modifying molecular genetics to develop personalized and targeted treatments based on RNA interference. Modification in RNA therapies utilized small RNA subunits in the form of small interfering RNAs (siRNA) for overexpressing the specific genes of, most commonly, breast, colon, gastric, cervical, and hepatocellular cancer. RNA-conjugated nanomaterials appear to be the gold standard for preventing various malignant tumors through focused diagnosis and delivering to a specific tissue, resulting in cancer cells going into programmed death. The latest advances in RNA nanotechnology applications for cancer diagnosis and treatment are summarized in this review.

## 1. Introduction

**Figure 1:**
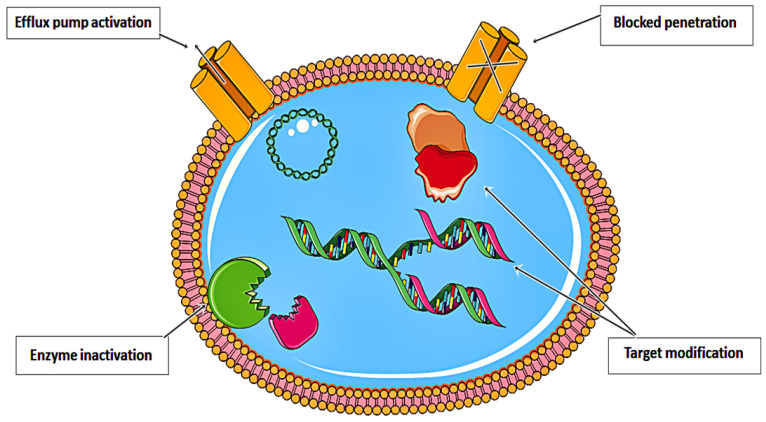
Resistance strategies developed by anticancer drugs that limit their therapeutic efficiency.

Despite decades of basic and clinical investigation, as well as trials of new therapeutic modalities, cancer remains a substantial cause of mortality worldwide [1]. Various resistance strategies have led to the near inefficiency of anticancer drugs. As illustrated in Figure 1, these drugs have been rendered almost ineffective. Recent approaches in nanomedicine have prompted the development of effective theranostic platforms for a myriad of biological and biomedical applications [2]. Nanomaterials (i.e., niosomes [3], polymer-based nanocapsules [4], nanoparticles (NPs) [5,6,7,8], metal nanocages [9], nanocomposites [10], nanoliposomes [11], and engineered nanohydrogels [12]), with highly controlled geometry and physic-chemical properties, have been introduced as promising tools for recognizing cancer tissues and also serve as novel drug delivery systems (DDSs) to achieve active targeting [2,13,14,15,16,17,18,19,20,21]. It is now believed that nanotechnology can purposefully improve the clinical outcome of cancer therapies through improving the tolerability of the efficacy of novel drugs [22] or delivering proteins, DNA, RNA, and various types of molecules to cancerous cells [23,24]. Several nanomaterial-based biosensors have also been developed for the accurate sensing of tumor markers [24,25].

Similarly, biodegradable and biocompatible natural and engineered biomolecules (including proteins, peptides, polysaccharides, and nucleic acids) have been broadly examined to synthesize nanostructures [26,27]. Biomolecule-based building blocks provide particular features that make it not feasible to be reproduced in these synthetic materials. However, multifunctional approaches can be developed by exploiting biomolecule-derived elements concerning cancer targeting and therapy [27].

It is scientifically established that nucleic acids can be manipulated and designed to create many nanostructures [28]. In the past, researchers have studied the preparation and characterization of DNA nanoparticles (DNA NPs), utilizing complex processes, such as coacervation [29,30]. In this respect, charge-neural biodegradable DNA NPs were synthesized by compacting a small-sized single DNA molecule and loading it on magnetic NPs [31]. This strategy is now considered a beneficial translatable gene therapy platform for overcoming challenging biological barriers by enhancing nuclear uptake across tiny nuclear pores of dividing cells and is being widely investigated to treat various conditions (i.e., malignancies and respiratory diseases) [31,32,33].

Due to its increased thermodynamic stability, the RNA structure can be more flexible while folding into different structures (i.e., rigid structural motifs), and it produces diverse building blocks for numerous therapeutic applications [34], including the fabrication of nanosensors and nanodevices [28]. Furthermore, the thermal stability of RNA also allows it to produce multivalent nanostructures possessing specific stoichiometry [28,35]. In this view, introducing novel methods for the re-assembling of RNA molecules has recently spurred interest in investigating the biomedical application of RNA nanostructures.

Nanomedicine has led to the development of a variety of nanoscale therapeutics and diagnostics to treat a variety of diseases, specifically cancer [6,14,15,17,18,36,37,38,39,40,41,42,43,44,45,46]. This fact is broadly exploited in the field of DNA nanotechnology [47]. Generally, methodologies for DNA nanotechnology can be applied to RNA nanotechnology [48]. Despite their similarities, DNA and RNA nanotechnology differ in a few key aspects. Inter-and intra-molecular interactions, as well as stem-loops, are abundant in the RNA molecules. These allow the formation of complex secondary and tertiary structures (i.e., bulges, stems, junctions, loops, etc.) and thus can be utilized to create ‘dovetail’ joints among the building blocks [35]. RNA can serve as a potential new therapeutic modality for cancer due to its lack of accumulation in vital organs [49].

In the past, numerous RNA-NPs have been prepared by an automated self-assembly process and studied in the context of cancer research [50,51]. A key challenge in programming RNA strands to assemble into nanostructures is to create a folding pathway to avoid kinetic traps [52]. In addition, by introducing chemical modification into nucleotides without substantial changes in RNA content, RNA-NPs will escape host RNA decay pathways. In addition, RNA nanostructures might have immunologic properties, making them valuable tools for in-vivo applications [49].

The conjugation of polymeric NPs with RNA molecules, such as RNA aptamers, cause these bio-conjugates to be easily absorbed by specific tumor cells, and therefore, can be considered as a beneficial strategy towards the controlled release of polymeric drug delivery vehicles [53]. In the growing field of RNA nanotechnology to fight cancer, RNA aptamers have attracted great attention as tools for delivering other RNA therapeutics, such as short interfering RNAs (SiRNAs), to specific organs [54]. Moreover, polyvalent RNA nanostructures have been effectively fabricated as carriers of siRNA, ribozyme, and anticancer agents to tumor sites [55].

Using in-vitro and in-vivo experimental models, Yin et al. exploited RNA-based technology to efficiently deliver anti-microRNA to cancer cells derived from breast tissue [56]. Ghimire and colleagues showed that RNA-NPs could be utilized as rubber for constraining vessel extravasation to improve the targeting of cancer cells and increase their renal excretion, thus reducing their toxic effects [57]. Recently, radiolabeled RNA NPs were developed for specific targeting and efficient tumor accumulation with desirable in-vivo biodistribution [58]. According to Kim et al., dual-targeting polymeric siRNA NPs were synthesized by multiple processes, including electrostatic deposition and poly-L-lysine condensation. Researchers found that these nanostructures are capable of efficiently delivering siRNA to tumor cells [59]. Xu et al. successfully delivered delta-5-desaturase via dihomo-γ-linolenic acid-loaded RNA-NPs for suppressing the growth of cancerous colon cells via the induction of apoptotic cell death [60]. Haque and colleagues systemically injected synergistic tetravalent RNA-NPs into the tail-vein of mice. They observed that these RNA nanostructures preserve their biological function within cancer cells without entering other tissues or organs [61].

RNA therapies provide new insights for cancer treatment. The escalating growth of RNA nanotechnology in cancer theranostics demands the preparation of an updated review. This article comprehensively discusses recent findings and highlights the promising avenues of RNA nanotechnology implemented to design stable RNA nanostructures for diagnostic and prognostic purposes. Finally, we will discuss strategies to improve and overcome many technical obstacles in this field.

## 2. RNA Nanotechnology for Diagnosis of Cancers

Biosensors are diagnostic systems that convert a natural response into a programmable signal [62]. The calculable signal can be electrochemical, optical, thermal, or piezoelectric. Low detection limits, accuracy, and high sensitivity make electrochemical biosensors the most reliable of all. Electrochemical biosensors have a great prospective in the real-sample analysis [63]. The combination of nanotechnology with biosensors is a hallmark for disease assessment and the planning of its cure. Nanoscience is the science of the molecular and atomic manipulation of materials. It entails creating and managing chemical, physical, and systems in sizes of 1–200 nm. Nanotechnology has many applications in the biomedical field, especially in medical imaging for disease diagnosis [64]. The exclusive physicochemical properties of NPs are used to develop biosensors of point-of-care accuracy, known as nanosensors. The performance of other electrochemical and enzymatic biosensors increases due to their small size as the distance between enzyme and electrode decreases. Some optical biosensors use noble metal NPs to improve optical properties and increase localized surface plasmon resonance (SPR); e.g., inter-particle plasmon coupling changes the color of NPs, which is used for improving the properties of biosensors grounded on the aggregation of NPs [65]. DNA oligonucleotides were the first nucleic acid-based NPs that served as the foundation of DNA origami technology, but nowadays, RNA oligonucleotide nanotechnology is an important alternative to DNA technology [49]. DNA and RNA have operational differences because of their different structural properties. RNA nanotechnology uses single-stranded oligonucleotides for designing diverse and functional RNA nanostructures. The specific structure and organization of functional groups in NPs make them an excellent tool for diagnosing and treating different diseases [66]. The main edge of RNA NPs includes therapeutic elements, regulatory moieties, and targeting ligands. The field of RNA nanotechnology is different from traditional RNA research, which targets 2D/3D structure-function relationships and intra-RNA connections, as it emphasizes mainly quaternary exchanges and inter-RNA interactions [51].

### 2.1. Benefits of RNA Nanotechnology in Targeting Cancer Treatment

RNA NPs are discrete entities, quite different from classical therapeutic RNA, including siRNA, anti-miRNA, miRNA, mRNA, ribosomal-RNA, and viral immune-stimulatory RNA. These traditional RNAs have a broad fundamental history of research in the RNA field. These small RNAs are caught by cells, and they stimulate RNA-sensing pattern recognition receptors (PRRs). Some of these are reported to be immunogenic.

There are a lot of benefits of RNA NPs as compared to traditional RNA, for example: (a) enhanced permeability and retention (EPR) effect, (b) the small size offers to promise pharmacokinetic and pharmacodynamic properties, (c) decreased liver accumulation, (d) Small non-coding RNAs or microRNAs serve as scaffolding and active elements in the bottom-up self-assembly of more complex nanomaterials, and (d) untraceable toxicity in-vivo [67]. Multinational characteristics of RNA nanostructures, such as targeting ligands and multi-drug loading, are useful for combination therapy [68]. RNA NPs show chemical, metabolic, and thermal stability in biological systems. The standardized volume of distribution V_d_, i.e., 1.2 L/kg of pRNA NPs, indicates the presence of a valuable amount in peripheral tissues, especially in a tumor. The comparatively small amount of clearance (Cl) value indicates the insufficient filtration of the NPs from the kidneys. A specific targeted delivery can be achieved by incorporating traditional RNA into NPs by taking advantage of these properties. These NPs show specific targeted delivery, higher therapeutic efficacy, and an increased in-vivo half-life [69].

Cancer nanotechnology faces a serious challenge of non-specific accumulation of delivered NPs in healthy organs, such as lungs, liver, spleen, and kidneys [70]. The non-specific accumulation leads to poor transport of NPs to the tumor site, unwanted side effects, and toxicity. The Guo laboratory combined targeting ligands and a sequence of pRNA-3WJ-based NPs for advanced targeted delivery. After systemic delivery in tumor-bearing mice, these RNA NPs are precisely transported to tumors within almost 4 h. These NPs remain at the tumor site for more than 24 h. After more than a few hours of injection, no or minimal organ accumulation was detected. Several common cancer models were used to get consistent results, such as breast, colorectal, prostate, glioblastoma, gastric, and head and neck cancers. The targeting ligands were altered based on the overexpression of specific targets in tumor tissues [71]. RNA with negative charge limits non-specific interactions with negatively charged cell membranes, which is important for high selectivity. The ratchet-like shape and strong elasticity of pRNA-3WJ-based NPs show improved EPR effects and higher tumor penetration. Overall, these findings showed that pRNA-3WJ-based NPs could be synthesized simply, with high selectivity and low side effects for healthy tissues. This promising bio-distribution is a significant signal of pharmacological profiles of RNA NPs [51].

### 2.2. Nano-Biosensors as Developing Trend in Cancer Diagnostics

**Figure 2:**
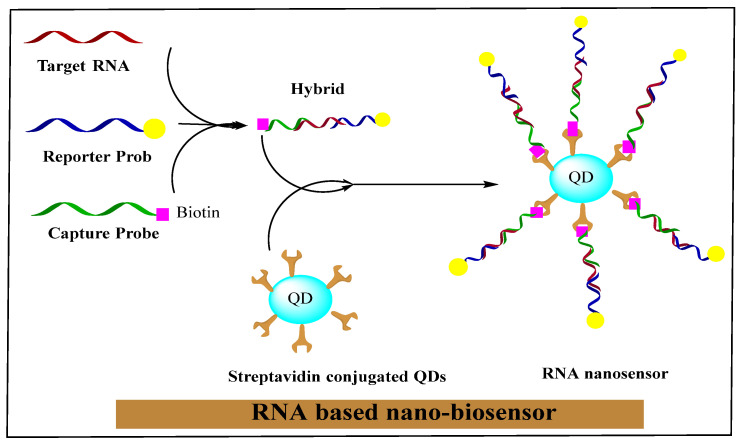
RNA-based nano-biosensor. Reprinted with permission from [75]. Copyright 2021 Elsevier.

The nano-biosensor is an innovative unit that creates nano-conjugated biological systems, which function as signaling mediators to detect the specific contents of medical, biochemical, or physical agents. The related data is transferred in the form of signals using thermometric, piezoelectric, optical, magnetic, electrochemical, and micromechanical methods. The signals produced by these methods depend on the bio-recognition of a cancer cells-related surface or intracellular biomarkers through bio-ligands or antibodies. In a new era of research, nano-biosensors will increasingly be classified based on bio-recognition and signal transduction elements. RNA-based nano-biosensors are displayed in Figure 2.

In the basic structure of a sensor, there are two main components: (i) the target analyte that can be a nucleic acid, antibody, drug, protein, or cell-surface molecule; and (ii) the transducer used for altering a signal into a form of energy. It can be detected electrochemically by detecting the energy it produces (voltage and current), (absorption and luminescence), and mechanically (resonance). The exclusive and tunable physicochemical properties of nanostructures, such as enhanced electric conductivity, greater area to volume ratio, high reactivity, unique magnetic properties, and powerful scattering and absorption, make them a fascinating tool for bio-sensing. The nanomaterials’ ability to interact effectively with biologically important analytes and convert those interactions into considerably enhanced signals has enabled a new class of early diagnostic procedures. Gold NPs (AuNPs) and QDs are excellent signal transducers because the existence of a specific analyte regulates the signal generated by the material’s optical properties. Nano and microsensors reduce the size of the receptor to improve their responsiveness. Increased signal-to-noise (S/N) ratio is responsible for this enhanced sensitivity. However, a running device’s success depends on its ability to detect a tremendously low concentration in a reasonable amount of time.

The overall sensitivities of a sensor are often affected by mass transport limitations, such as when the substance is transported to the receptor by diffusion mechanism. The limiting factor in saturating the receptor for a specific geometry of the sensor is the time required by the analytes to reach the sensor area. As the receptor area becomes smaller, the time grows higher. The decreased number of analytes obstructs the detection of extremely dilute solutions. Many practical solutions have been tested to concentrate the dilute solution straight onto the sensor surface. For example, Melli et al. formulated a unique solution with a series of micropillars with superhydrophobic surfaces to simplify the management of samples with a low concentration of analytes. A diluted solution can be placed on these micropillars, the target molecule will become concentrated by the evaporation of liquid, and the detection time is significantly decreased [72].

Halo et al. used the same theory to detect colorectal cells by mRNA quantification. They developed a NanoFlare platform comprising a single layer of single-stranded DNA (ssDNA) coated on spherical AuNP. A fluorescent reporter was added to a short DNA counterpart that was hybridized to the ssDNA recognition sequence. The reporter fluorophore was slaked while it was close to the AuNPs, but it broadened when the target mRNA displaced the DNA, allowing the fluorescence reading. The detection limit of the nanosensor was about 100 cancer cells/mL blood [72].

A particular type of RNA aptamer that causes small-molecule fluorophores to emit fluorescence is called a light-up aptamer. Several new light-up aptamers have been developed that can bind to different biocompatible fluorogenic ligands and make their way to the design of modern RNA-based molecular strategies for sensing applications. The Systemic Evolution of Ligands by EXponential enrichment (SELEX) is a combinatorial procedure for identifying such aptamers. RNA aptamer libraries were exposed to recurrent cycles of collection and amplification in this procedure, resulting in RNA aptamers having the strongest selectivity for the target ligand. Various RNA NPs can be coupled with light-up aptamers having programmable sensing capabilities to create dynamic reporting entities [73].

### 2.3. RNA Nano-Biosensors

RNA/DNA nano-biosensors can measure the responses produced by aptamer hybridization or nucleic acid conversion processes as promising diagnostic tools [74]. As aptamers, RNA or DNA are single-stranded nucleic acid oligomers whose structure is extremely organized and complex, forming a strong connection with the target molecules (Figure 1) [75]. The RNA with a functional group usually reacts with molecular labels with an orthogonal reactive group in a suitable reaction environment. Fluorescent compounds activated by NHS are attached directly to an NH_2_ group of the RNA fragment, and similarly, thiol to maleimide and alkyne to azide, etc. RNA was directly manufactured, and classical pairing reactive groups, e.g., amino –NH_2_-COOH, azide, maleimide, alkyne, and thiol, were incorporated by similar methods for the production of polyvalent RNA NPs [76]. Single-stranded RNA NPs that are used for many purposes, such as biodistribution and diagnostic studies, are the subsequent derivatives of fluorescent dyes (FITC, Cy5, Cy3, and AF-647) attached to RNA [77].

RNA polymer is conjugated with NPs (such as iron oxide NPs, quantum dots, and gold NPs), and a sequence of research studies used siRNA, pRNA, and phi29 for this purpose. The siRNA was coupled with many nano-based imaging agents to form multifunctional NPs exhibiting diagnostic and therapeutic moieties.

Bhatia et al. established tumor-highlighted peptides (F3) and siRNA coupled to the PEGylated QDs core as a framework. The F3 peptide was conjugated to amine group-modified QD via sulfo-LC-SPDP (sulfosuccinimidyl 6-(3′-(2-pyridyldithio)-propionamido) hexanoate as a heterofunctional cross-linker, and thiol modified siRNA used sulfo-SMCC (sulfosuccinimidyl 4-(N-maleimidomethyl) cyclohexane-1-carboxylate) for conjugation. The QD-siRNA/F3 conjugate NPs were proficiently transported to HeLa cells and unconfined from their endosomal setup, which provided the demolished EGFP signal. These siRNA-NPs conjugates exhibited both imaging and therapeutic properties. Furthermore, the siRNA was coupled to iron oxide NPs, which showed magnetic characteristics for biomedical applications. The linkage of siRNA to iron NPs exhibited a double response, such as the in-vivo delivery of siRNA and gathering of siRNA in the tumor by MRI and the near-infrared fluorescent (NIRF) in-vivo optical imaging. The amine groups of iron oxide NPs were treated with m-maleimidobenzoyl N-hydroxysuccinimde ester (MBS) for linkage between siRNA and magnetic NPs. After that, the reduced thiol group of RNA was treated, and magnetic NPs were coupled with and near-infrared Cy5.5 dye and membrane translocation peptides. The MRI and NIRF were used simultaneously to see the siRNA-magnetic NP uptake. The coupling of AuNPs with RNA is studied to enhance the accessibility of tethered RNA splicing enhancers. Guo et al., in 2007, produced a pRNA of the phi29 and DNA-packaging motor linkage to AuNPs to study the phage assembly. In this case, the SH-labeled DNA oligonucleotide was merged with 3′ terminal of pRNA to incorporate the thiol (-SH) group into pRNA. Then, the thiol-treated pRNA was conjugated to gold NPs. The pRNA/AuNPs conjugate was attached to procapsid by an in-vitro phage assembly. Guo’s group proved that the RNA polymer was coupled with the AuNPs effortlessly, and this procedure can be used for imaging purposes. Currently, pRNA-3WJ was coupled straight to the quantum dot for resistive biomemory applications. They introduced a sephadex G-100 resin-recognizing RNA aptamer in the biotin-labeled pRNA/3WJ (SEP_apt_/3WJ/Bio) theme for the conjugation of pRNA-3WJ to the quantum dot (QD). Firstly, the SEPapt/3WJ/Bio was attached to G-100 resin, and then the streptavidine-labeled quantum dot was linked to SEP_apt_/3WJ/Bio by streptavidine-biotin coupling on Sephadex G-100 and STV/QD-SEP_apt_/3WJ/Bio conjugates were subjected to dissociation in the elution buffer. Later, the STV/QD-Bio/3WJ_b_, 3WJ_a,_ and SEP_apt_/3WJ_c_ were split by elution buffer. After that, the STV/QD-Bio/3WJ_b_ fragment was filtered and reconvened with pRNA 3WJ_a_ and thiol-labeled pRNA 3WJ_c_ for resistive biomemory application. Hence, the RNA polymer can be easily incorporated with other NPs and used for various diagnostic, therapeutic, and bio-electronic fields [77]. Table 1 summarizes the role of RNA-based nanostructures in diagnosing cancers.

**Table 1:** Summary of RNA-based nanostructures in diagnosis of cancers.

RNA-Based Nanoparticles	Key Feature
Immune-Magnetic Exosome RNA (iMER)	Exosomal analysis of glioblastoma multiforme (GBM).
Anti-RNA aptamer	Initial detection and analysis of residual GBM.
RNA tetrahedrons	Target triple-negative breast cancer cells.
Oligonucleotide-treated Au-NPs	Analyzing circulating tumor cells (CTCs) of the prostate.
miR-122 mimicked using cationic lipid NPs	Theranostic agent against hepatocellular carcinoma.
Superparamagnetic iron oxide NPs (PEG-g-PEI-SPION)	Initial detection and analysis of gastric cancer.

Graphene consists of a single-atom dense two-dimensional honeycomb framework made of sp^2^-bonded carbon atoms. Many graphite structures, such as nanotubes, graphite, and fullerenes are produced using graphene. British scientists Andre Geim and Konstantin Novoselov were awarded Nobel Prize in Physics in 2010 at the University of Manchester for their revolutionary research on graphene.

Presently, novel functional materials can be manufactured, and siRNA can be delivered to cancer cells by immobilizing RNA on graphenes. Hu et al. produced polydisperse and stable RNA-graphene oxide nanosheets by covalently immobilizing an RNA aptamer on grapheme oxide. Sharifi’s group exfoliated graphene flakes from nano-crystalline graphite to yield conducting and transparent RNA-graphene-labeled thin films by using RNA as a surfactant. These thin films are used in a lot of electronic applications [77]. Proteins and peptides are used to form nucleic acid carriers because the nucleotides are shortened by electrostatic linkages with positively charged amino acids of proteins, which are used to transport nucleic acids and small non-coding RNA molecules. On the other hand, the amino acids impart bioreversible polyplex stabilization of the system, endosomal escape, and targeted delivery [78].

### 2.4. RNA Nanotechnology in Diagnosis of Different Cancers

#### 2.4.1. GBM

Shao et al. developed a microfluidic platform entitled immune-Magnetic Exosome RNA (iMER) for the exosomal analysis of GBM. Three functional sections were combined by iMER, i.e., real-time RNA analysis, targeted up-gradation of extracellular vesicles, and on-chip RNA isolation. The up-gradation or enrichment process detached the cancer exosomes immunomagnetically from host-derived exosomes, and the later analysis was executed on enriched populations. Later, a glass-bead filter was used to pass the lysate collected after the lysation of exosomes. RNA was immobilized onto glass beads by forming electrostatic bonds during this method and then extracted and quantified by qPCR. Magnetic microbeads with anti-epidermal growth factor receptor (EGFR) antibodies were used to identify and enrich GBM-derived exosomes. These GBM-derived exosomes were incubated with beads, and the whole surface seemed thickly covered. A large quantity of mRNA was found in these vesicles, having mRNA of nuclear proteins as well [72].

Spherical nucleic acids SNA are a type of NPs that are made up of a NP core treated with oligonucleotide structures and comprised of RNA interference RNAi reporter molecules and therapeutics. The exclusive 3D SNA structures are resilient to nuclease degradation and co-opt into the cells despite the lack of transfection agents. SNAs act as diagnostic agents and have the potential to detect two unique mRNA sites at a time inside of a cell [77]. These SNAs can effectively cross blood-brain and blood–tumor barriers and are broadly experimented against the most violent and widespread type of malignant brain cancer, i.e., GBM models [79]. Currently, much attention has been given to EGFRvIII, which is an EGFR receptor variant and is associated with GBM progression. DNA and RNA aptamers have been fabricated and used for GBM detection by the involvement of EGFR.

Iqbal et al. sequestered an anti-RNA aptamer from purified human protein by creative selection. The authors confirmed the ‘aptamer’s ability to detect and seize murine and human GBM cells after its immobilization on a glass substrate. Aptamer binds with wild-type and mutant EGFR with excellent specificity and affinity (Kd = 2.4 nM). This technique is used for the initial detection and analysis of residual disease. Similarly, Iqbal’s group fabricated a flow-through lab-on-chip tool that used surface-attached aptamer’s affinity for GBM’s overexpressed biomarker, i.e., EGFR, to prove that a microfluidic-based technique can be used to detect and capture GBM cells. Later, the same group took radical steps in diagnostics related to anti-EGFR aptamers and came up with two succeeding articles on tracing the differential dynamics of GBM cell structure on substrates grafted with aptamers. They analyzed the dynamic morphology of GBM in computational single-cell metrics to identify and capture tumor cells [80,81].

Choulier et al. linked cell-SELEX and protein to separate RNA aptamers. These aptamers have the potential to bind specifically to integrin α5β1, which is an αβ heterodimeric receptor related to cancer angiogenesis and GBM ferociousness. The authors used in histo-fluorescence analysis on patient-based xenografts and a fluorescence-related analysis on cell lines to verify the diagnostic capability of tagged aptamers [82]. Recently, some specific aptamers were considered as markers for metastasis and recurrence of GBM stem cells (GSCs) and brain tumor-initiating cells (TICs).

Rich et al. designed a pool of DNA aptamers distinguishing TICs with an extremely low dissociation constant (Kd between 0.12 and 3.75), and an aptamer-Cy3-related fluorescence proved the binding. In 2019, Affinito et al. used an RNA library on basic human GSCs to design a 20-F-RNA aptamer A40s, which was explicitly related to the GBM cells. The authors established the detection of A40s-based analysis in GBM cells and GSCSs of human tissue sections [83]. Here, the aptamer showed a great affinity (Kd = 41.92 nM) for target cells in a less nanomolar range. As stem cells play an important role in chemo-resistance and metastasis, these aptamers can be used in the clinical field to detect violent areas and analyze GBM treatments [81].

#### 2.4.2. Breast Cancer

Currently, three-dimensional RNA NPs with tetrahedral structures have been designed. RNA tetrahedrons are used in various applications in nanomedicine and nanomaterials because of their structural permanence and mechanical rigidity. The EGFR aptamer was bonded with the RNA tetrahedron structure to target triple-negative breast cancer cells. After IV administration, the drug-loaded NPs face a sequence of biological blockades. NPs face quick opsonization and succeeding sequestration by local macrophages under physiological conditions. As a result, healthy organs, such as the spleen and liver, accumulate very high levels of NPs [84]. To address these issues, RNA NPs were used to specifically target tumor cells and avoid renal clearance and organ accumulation. For example, Prna-3WJ NPs were fixed with an RNA aptamer specific for EGFR. These NPs specifically targeted triple-negative breast cancer (TNBC). Fluorescence confocal microscopy was used for the histological analysis of tumors to detect the precise directing and retention of RNA NPs in a cancer-frozen cross-section. Both treated and control groups were compared, and it became evident that pRNA-3WJ-EGFR showed extraordinary accumulation at tumor cells without disturbing the healthy organs [69].

In another study, QD-mi RNA let-7a-gold NPs (QD-RNA-Au NP) were conjugated with Chitosan-based nano-formulation, including negatively charged poly (g-glutamic acid) (PGA) for transfer to breast cancer cells. In the cells, the QDs were separated by dicer-mediated release and showed fluorescence for theranostic applications [85].

Mediley et al. described the use of an AuNP aggregation-related colorimetric sensor for straight cancer cell detection. However, the signal produced by aptamers after binding with cancerous cells was too low to detect CCRF-CM cells due to their weak binding affinity. To solve this problem, Lu et al. used S6 RNA aptamer-linked multifunctional oval-shaped AuNPs and the monoclonal anti-HER2/c-erb-2 antibody for multivalent attachment of AuNPs with target cells for extremely sensitive analysis of SK-BR-3 breast cancer cells. AuNP-linked colorimetric techniques are used for initial and sensitive in-vitro recognition of cancerous cells [86].

#### 2.4.3. Prostate Cancer

Sioss et al. fabricated a nanowire-resonator array sensor for signal detection using oligonucleotide-treated AuNPs to analyze RNA in circulating tumor cells (CTCs). In this method, RNAs were attached to AuNPs previously immobilized on the sensor by hybridization. The resonance frequency of the sensor was changed by AuNPs after adding mass to it. The authors calculated this change in resonance frequency and detected PCA3 RNA, a nucleic acid prostate cancer marker. After measurement of RNA and volume used, they calculated that the level of development was 1 CTC/10 mL blood. The sensors used were extremely sensitive and specific, showing a single-nucleotide inconsistency calculation. AuNPs have a large surface area, and they are used as a support to increase signal formation by enhancing molecular binding events [87].

The prostate-specific antigen (PSA) was spotted in prostate cancer cells and the neovasculature of many types of malicious neoplasms, such as breast cancer [88], lung [89], and some other tumor cells [90]. One study reported ligand-receptor relation of RNA NPs with PSA aptamers. The 3WJ-RNA NPs were attached to PSA aptamer (PSA-RNA), indicating extraordinary tumor accumulation in the bio-distribution analysis [77].

Mohamadi et al. fabricated a microelectrode biosensor based on the PSA mRNA and magnetic NPs-based circulating tumor cells. Another study demonstrated aptamer-based in-vivo targeting by pRNA-3WJ NPs containing the anti-prostate-specific membrane antigen (PSA) RNA aptamer. On the other hand, folate can be coupled with pRNA-3WJ NPs as an innovative strategy for improving nanocarrier distribution.

The pRNA-3WJ-folate conjugates have been used in many cancer cells in which FR is overexpressed, such as colorectal, gastric, head and neck cancers, and glioblastoma. The in-vivo distribution of RNA NPs was studied using pRNA-X NPs containing fluorophore or folate. These NPs were injected into athymic mice with KB cells xenografts. Whole-body images were taken at different time intervals and indicated the high accumulation of RNA NPs in cancer cells within 4 h. Specific localization of pRNA-X NPs in the tumor was calculated at the 8th hour of organ imaging, without accumulating in healthy organs [69].

#### 2.4.4. Liver Cancer

Cationic lipids (CLs) are used for liposome-oriented DNA/miRNA transport. These lipids are made up of a linker to which a hydrophobic part is linked to a cationic head part. The positively charged head group is attached to the negatively charged phosphate group of nucleic acids [91]. Liposomes have advantages of less risk of immunological reaction, less toxicity, and are easy to handle, making them a useful tool for non-viral drug delivery and diagnostics. Lipid NPs containing lactosylated gramicidin were exploited to transfer anti-mir-155 to hepatocellular carcinoma. Hsu et al. transferred miR-122 mimic using cationic lipid NPs to suppress miR-122 in hepatocellular carcinoma [92].

#### 2.4.5. Gastric Cancer

Chen et al. developed an MRI-visible system based on superparamagnetic iron oxide NPs (PEG-g-PEI-SPION) and polyethylenimine conjugated to polyethylene glycol (PEG) for the delivery of siRNA to gastric cancer. Despite its use in cancer gene down-regulation, this PEG-g-PEI-SPION has verified itself as an extremely effective contrast agent for in-vivo MRI scanning as well. In the same way, Sun et al. designed Micro-RNA-16-loaded magnetic NPs to solve drug resistance challenges in the mouse gastric cancer model. Polyethylene glycol (PEG)-coated iron oxide (Fe_3_O_4_) NPs were used in this study. These magnetic NPs showed highly efficient in-vivo imaging along with increased (human gastric cancer cell line 7901) SGC7901 sensitivity to the drug Adriamycin [93].

Rychahou et al. developed the precise delivery of folate-linked pRNA NPs into colorectal cancer. After IV injection, the RNA NPs showed accumulation in metastatic cells of colorectal cancer, lung, liver, and lymph node cancer, but no accumulation was found inside healthy organs [69].

## 3. RNA-Nanomaterials for Targeted Therapy of Different Cancers

**Figure 3:**
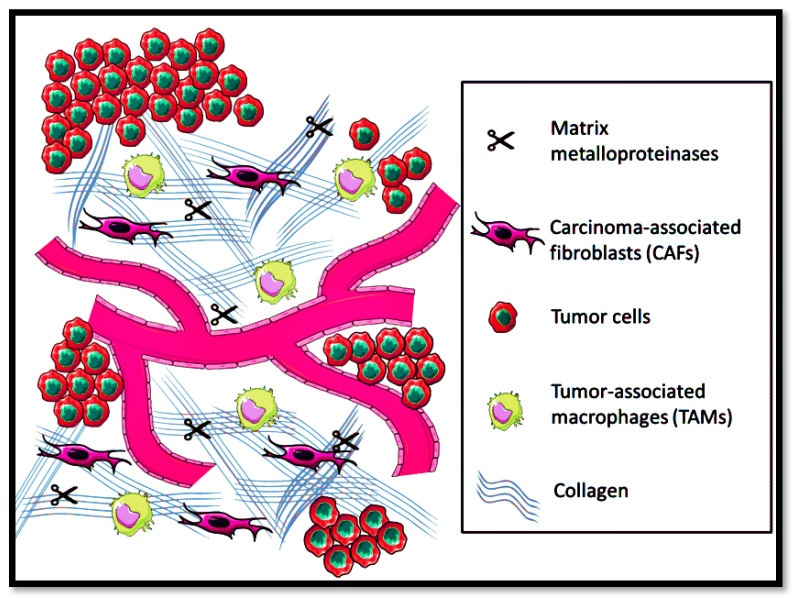
Tumor microenvironment prevalence in the extracellular matrix.

Extracellular matrix (ECM) is composed of glycoproteins, elastin, collagen, and hyaluronic for providing solid structural support for cellular processes, i.e., proliferation, cell migration, and growth [94]. Moreover, ECM also systematizes the intracellular communication of cytokines and growth factors and acts as a source of physical barrier against the tumor microenvironment [95]. However, in solid metastatic tumors, the ECM equilibrium in maintaining homeostasis gets affected, leading to disorganization of the physicochemical and biochemical features, as shown in Figure 3.

Conventional cancer treatment has failed to mitigate the impact of malignancies [96]. Therefore, multi-functionalized NPs were preferred to be synthesized for targeted action via attachment of specified ligands to target the tissues that are highly over-expressed in certain diseases. The development of targeted and personalized therapeutics based on RNA interference has also been made possible due to advances in molecular genetics [97]. The modification in RNA therapies utilized small RNA subunits in the form of small interfering RNAs (siRNA) for overexpressing the specific genes of the related cancers [98]. The RNAi technology can be exploited to change the oncogenic characteristics of breast cancer cells, making them highly conducive to apoptosis cell death. Combination therapies are useful approaches to generating apoptotic effects mediated via carcinogenic pathways and help to overcome drug resistance [99].

**Figure 4:**
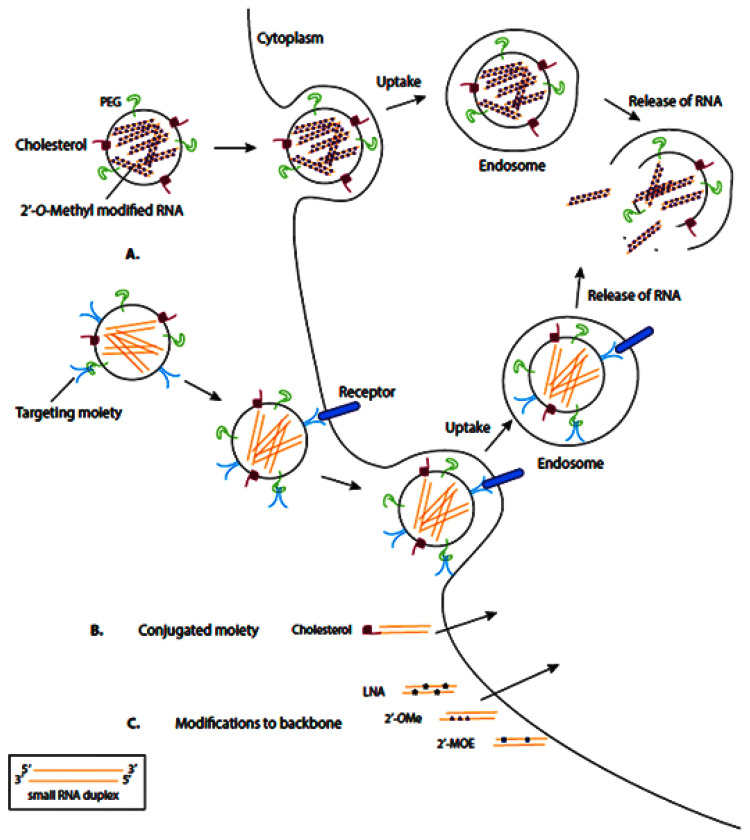
Schematic representation of common delivery methods for RNA-based therapies. LNA: locked nucleic acid (2′4′-methylene; 2′OMe: 2′-O-methyl; 2′MOE: 2′-O-methoxyethyl. Nanocarriers can enter the target cells via endocytosis (**A**), direct conjugation to moieties (**B**), and chemical modification (**C**). Reprinted from [100].

RNA-based drugs are often entrapped or attached to the surface of different nanovehicles to deliver the cargo to the cells. These nanovehicles can be modified with various moieties, including polyethylene glycol (PEG) and cholesterol, which enhance them by the membrane of target cells, where the nanovehicle can enter the target cells via endocytosis (Figure 4A). Meanwhile, some RNA-based therapeutics are conjugated to moieties directly, which facilitates their transmembrane transport (Figure 4B). In another therapeutic approach, the synthesized RNA-therapeutics are chemically modified to enhance their binding affinity, stability, and biocompatibility (Figure 4C).

**Figure 5:**
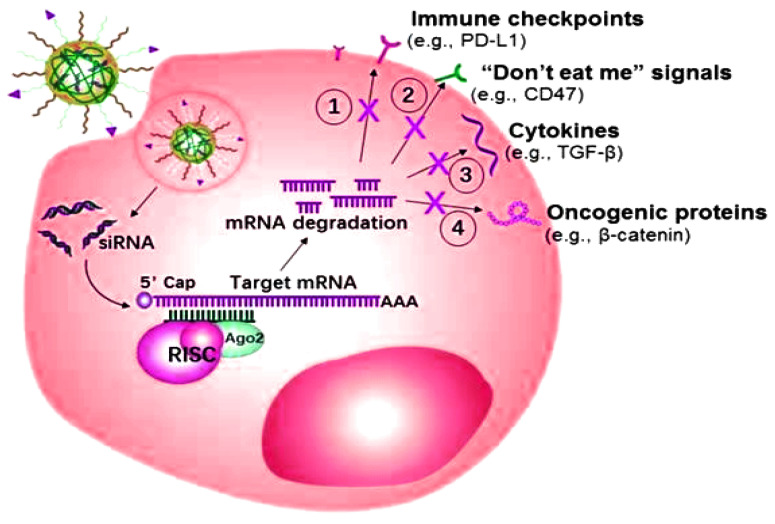
Small interfering RNAs (siRNA) mechanism of action for overexpressing the specific genes of the related cancers.

The miRNA or siRNA possesses the capability to bind with the enzyme-containing molecule RNA-induced silencing complex (RISC), resulting in the enzymatic cleavage of the target mRNA [101]. Targeted mRNA has the capability of silencing over-expressed genes as well as inducing programmed death-ligand 1 (PD-L1) efficacy towards programmed apoptosis against the deadliest cancer cells, as shown in Figure 5. The targeted role of mRNA as a ligand in various cancers is shown in Table 2.

**Figure 6:**
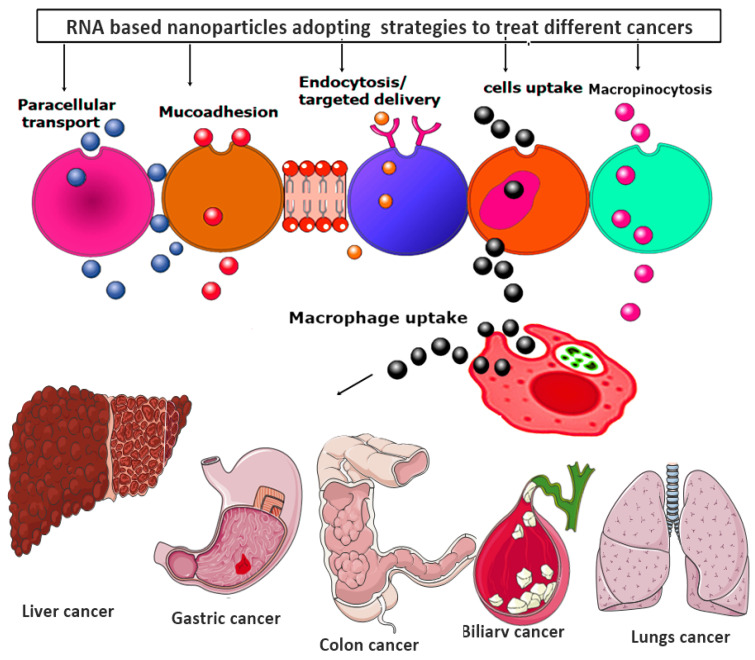
RNA-based nanoparticles various advanced strategies for providing the targeted and efficacious treatment against various metastatic cancers.

Moreover, all the RNA-based nanoparticles tend to adopt various advanced strategies to provide the targeted and efficacious treatment against various metastatic cancer, as shown in Figure 6.

### 3.1. RNA NPs

Cancer of the breast is the deadliest disease in the world [109]. Breast cancer therapy includes radiation therapy, chemotherapy, adjunct therapy (radiation therapy and chemotherapy), endocrine, and ligand-mediated therapy [110]. Guo et al. (2020) [102] conjugated PTX with RNA via the synthesizing prodrug for PTX as PTX-N3. PTX-N3 prodrug was synthesized by admixing the PTX, and other substituents in a 20 mL DCM solvent. The reaction mixture was then stirred at room temperature for 36 h following filtration and rotary evaporation to attain the yield’s crude product. The crude product was then purified by silica gel chromatography using n-Hexane: ethyl acetate as an eluent. RNA-6 alkynes oligomers were synthesized via a stranded solid-phase RNA synthesis and purified through desalting. RNA sequences of nine types were isolated and conjugated to PTX using copper (I)-catalyzed alkyne-azide cycloaddition by click addition. The reaction mixture was then diluted with ethanol followed by overnight incubation for RNA precipitation in DEPC-H_2_O. The precipitated were re-dissolved and purified by the ion-pair reverse phase HPLC in a PTX-labeled RNA for assembling NPs. Finally, an RNA four-way junction NP (4WJ-X nanostructure) with ultra-thermodynamic stability to solubilize and load PTX for targeted cancer therapy was developed by a 3D computational model generated using Swiss PDB Viewer and PyMOL Molecular Graphics System. Assembly of NPs was confirmed using a specified buffer solution. EGFR NPs were also conjugated with one of the aptameric RNA oligomers and 4WJ-X-24 PTXs. It was observed that each RNA NPs was found to be successfully attached via covalent interaction to twenty-four molecules of PTX as a prodrug. The developed RNA-PTX complex was found to be structurally stable and rigid. It was concluded that RNA NPs proved to help increase the water solubility of the BCS class II drug PTX by 32,000-fold, which possesses the issue of low water solubility. This treatment strategy of RNA functionalization in the form of intravenous injections resulted in the specified cancer targeting. RNA ligand proved to dramatically inhibit the growth of breast cancer with non-detectable toxicity and immune responses in mice. Moreover, no mortalities were observed at the LD_50_ dose of PTX [102].

### 3.2. Nanotechnology for Transfer of Therapeutic RNAs

By using nanotechnology for RNA, we are able to overcome many shortcomings of naked RNA molecules, such as poor chemical stability, easy degradation by nucleases, and extremely short half-lives for in-vivo applications. NPs act as a multipurpose and targeted system for the safe transfer of naked RNA molecules. The NP-based protect RNA from enzymatic cleavage and immune system threats. Because of their EPR properties, these nanostructures facilitate excellent RNA accumulation at the tumor site. Nowadays, the nanocarriers used for RNA delivery are lipid-based nanosystems [111], polymeric nanomaterials, bio-inspired nanovesicles, and inorganic NPs [112].

RNA NPs can easily include targeting ligands, counting RNA aptamers, and chemical ligands to impart specific targeting against proteins, fluorescent chemicals, and cell surface receptors. Chemical ligands, e.g., folate, are attached to the end of the RNA strand while the RNA is synthesized by modified RNA phosphoamidites. These ligands can also be attached to the RNA strand after RNA synthesis by the chemical conjugation method. The RNA aptamer strand, such as EGFR aptamer, can be manufactured by elongation of the scaffold strand.

The binding efficiency in-vitro and in-vivo of RNA NPs with ligands is tested by labeling them with radioisotopes and fluorescent dyes [113]. The labeled RNA NPs are incubated with cells for in-vitro binding and internalization studies. After incubation, these NPs are quantified by flow cytometry or fluorescence confocal microscopy [77]. Folic acid, FA, has attracted much attention for targeted siRNA delivery due to its small size, outstanding in-vivo stability, low immunogenicity, high binding affinity for folate receptors FRs, and great specificity to cancer cells. Folate receptors play an important role in diagnosing and therapeutics of inflammatory diseases and carcinoma. Folate receptors are overexpressed in cancer cells, and folic acid-decorated siRNA transporters bind precisely to FRs. The site-specific distribution of FAsiRNA conjugates and enhances siRNA concentration at the target site [112,114].

Folate receptors are overexpressed in epithelial cancer cells surfaces, permitting the folic acid conjugated NPs to target these cancer cells at a frequency higher than the normal cells.

RNA NPs can efficiently target cancer metastasis that is difficult to target because of the spread of cancerous cells to far-off cells and lymph nodes. Folic acid was used as a targeting agent by RNA NPs to simultaneously target colon cancer cells in the chief sites of metastasis, such as lungs, liver, and lymph nodes [49]. The 3WJ-based design procedure was used to form highly branched RNA dendrimers. RNA dendrimers used pRNA nanosquare as a symmetrical core for their formation. The formation of higher-ordered structures may face steric hindrance that is reduced by the square shape. Targeted delivery of NPs highly lessens the off-target toxicity and accumulation of NPs in healthy organs. RNA aptamers are an emergent field of therapeutics in which single-stranded RNA sequences form 3D structures by folding up and binding to extracellular domains of cell surface receptors with high selectivity and affinity. Nowadays, cell surface receptors are targeted by tens of RNA aptamers, such as prostate cancer (e.g., PSA), colon cancer (e.g., EpCAM), ovarian cancer (e.g., E-selection), glioblastoma (e.g., EGFRvIII), lymphoma (e.g., CD19) and breast cancer (e.g., EGFR, HER2, HER3) [49].

### 3.3. Small Interfering RNA-Selenium NPs

Small interfering RNA (siRNA) showed great potential in advanced therapeutics because of its highly sequential ability for silencing genes [115]. One of the most common causes of death in women is cervical cancer [116]. As a result, the right medication is necessary to minimize the severity of this cancer. For this purpose, Yu Xia synthesized biocompatible selenium NPs (SeNPs) and loaded them with the arginyl glycyl aspartic acid (RGD) Fc peptide for the sake of active targeting [103]. The RGDfC peptide bore a rich cationic charge and functionalized SeNPs for enhanced gene delivery. RGDfC-SeNPs have the capability of binding with HeLa cervical cancer lines.

Furthermore, Derlin 1-siRNA can be adjunct to the formulated conjugate of RGDfC-SeNPs via electrostatic interaction. The RGDfC-Se@siRNA successful conjugation and synthesis was confirmed via size determination by a zeta sizer, transmission electron microscopy (TEM), and Fourier transform infrared spectroscopy (FTIR). Elemental compositions of RGDfC-SeNPs were studied by energy dispersive spectroscopy (EDS). RGDfC-Se@siRNA characterization results proved that it followed Clathrin-mediated endocytosis for specifically reaching HeLa cancer cell lines and exhibited triggered siRNA release in a tumor microenvironment as compared to the biological microenvironment. However, in qPCR and Western blotting assays, both techniques showed eminent chances of gene silencing in HeLa cells. RGDfC-Se@siRNA was found to suppress the tumor invasion and division in HeLa cells via triggering the apoptosis pathway. Moreover, another crucial mechanistic approach of mitochondrial membrane disruption and reactive oxygen species generation (ROS) in HeLa cells was quite convincing in understanding that mitochondrial dysfunction mediated by ROS might play a significant role in RGDfC-Se@siRNA-induced apoptosis. Interestingly, advanced nanotherapeutics also presented substantial antitumor activity in a HeLa tumor-bearing mouse model [103].

### 3.4. siRNA-Polymeric NPs

Prostate cancer malignancy is one of the significant reasons for mortality worldwide. Prostate cancer is a non-skin malignancy causing the second biggest number of deaths in men when differentiated with different tumors. Conventional therapies are expected to cause erectile dysfunction, libido, obesity, and bone mass loss. Nanotechnology has modernized the field of medication to sidestep conventional therapies against metastatic cancers and different intracellular infections [117]. Xiao ding Xu et al. (2017) [106] synthesized siRNA multi-functionalized enveloped NPs for prostate cancer advanced therapy. SiRNA was self-assembled in the NPs via utilizing the library of oligo arginine and sharp pH-responsive polymers. Moreover, siRNA-functionalized and self-assembled NPs resulted in prolonged blood circulation, and the pH triggered a drug release through the activation of the endosomal membrane penetration. Furthermore, modification of the synthesized nanocarrier was done via attaching a specified molecular ligand that can recognize the PSA receptor. Synthesized nano-enveloped particles were characterized for size and zeta potential via DLS. The morphology of NPs was determined by TEM, fluorescence intensity, encapsulation efficiency, in-vitro siRNA release, luciferase silencing, endosomal escape, flow cytometry, in-vitro PHB1 silencing, digestion assay, Western blot, immunofluorescence staining, in-vitro inhibition of cell proliferation, a xenograft tumor model, and pharmacokinetic studies. siRNA multi-functionalized nano-enveloped carriers have the capability to strongly silence target genes expressions as well as strongly pre-dominant genes, such as prohibitin 1 (PHB1), resulting in significantly culminating tumor growth. Moreover, these advanced NPs also possess great potential for a robust siRNA delivery vehicle for prostate cancer-targeted therapy [106].

### 3.5. siRNA-Superparamagnetic Iron Oxide NPs

Globally, death from gastric cancer accounts for the majority of deaths. Conventional treatments have not been able to alleviate the consequences of this deadly disease. The development of targeted therapies for gastric cancer requires new technologies [118]. The most novel approach being considered is finding the primary targets, such as immune checkpoints such as PD-L1, in gastric cancer [119]. PD-L1 presents an over-expression on the activated T cells, resulting in the endosomal escape by cancer cells. The mechanism of inhibiting cancer cell regulation via PD-L1 cells lies in promoting and maintaining the T-cell responses in a controlled manner [120]. Xin Luo promoted siRNA delivery system for knocking down PD-L1 by developing folic acid (FA) and disulfide (SS)-polyethylene glycol (PEG)-conjugated polyethylenimine (PEI), complexed with superparamagnetic iron oxide Fe_3_O_4_ NPs (SPIONs) [107]. SPIONs were encapsulated with FA-PEG-SS-PEI via a ligand-exchange method, and then this conjugate was combined with synthesized siRNA-complexed cationic micelles. Furthermore, synthesized NPs were characterized based on binding capability, cytotoxicity, cellular internalization, and transfection efficacy. Cell viability assays demonstrated negligible toxicity and maximum cellular uptake as well. Cellular magnetic resonance imaging (MRI) presented that the NPs depicted maximum contrast for the T2 weight for cancer MRI. Furthermore, PD-L1 siRNAs displayed nominal knockdown of PD-L1 in the PD-L1-overexpressing gene. However, the co-culture model of activated T cells and the over-expressed gene cells represented an increased level of secreted cytokines. Therefore, these findings highlight the potential of this class of multi-functionalized polyplexed NPs for effective targeted PD-L1-knockdown therapy and diagnosis in gastric cancers, thus favoring the best theranostic approach [107].

Hepatocellular carcinoma (HCC) is the most common malignancy of the liver and the most common cause of morbidity and mortality [121,122]. A few molecular targeting drugs, such as sorafenib (SO), have been approved for advanced HCC, which also show a peripheral survival chance compared to conventional therapeutics. Unfortunately, its efficacy for HCC patients stayed substandard. Thus, the development of new methods for diagnosing and managing HCC is of the utmost importance. Gene delivery is the most preferred therapy involving RNA interference for the purpose of post-transcriptional gene silencing. Zhuo Wu et al. (2017) [105] developed a new class of amylose NPs, where the cationic amylose was used as the backbone functionalized with folate for targeting. Furthermore, SPIONs were utilized for the purpose of imaging and diagnosis for delivering specified surviving siRNA to hepatocellular carcinoma cells. The synthesized siRNA and multi-functionalized NPs were characterized based on zeta sizing, NMR, FTIR, cytotoxicity studies, cellular uptake, gene silencing, apoptosis signaling, and magnetic resonance imaging (MRI), and the results of these characterization techniques assured the successful conjugation of a new class of amylose NPs for the targeted delivery to HCC cells. Moreover, the novel nanocarrier system was able to initiate a specified and safe cellular uptake with increased transfection efficacy, promoting the downregulation of HCC cells. The resulting conjugate biocompatible complex based on cationic amylose could be used as a well-organized, prompt, and innocuous gene delivery vector. Furthermore, upon SPION addition, it holds great potential as a theranostic carrier for the gene therapy of HCC [105].

### 3.6. RNA-Mesoporous Silica NPs

Timely diagnosis and therapy are the prime responsibility of the healthcare system [123]. Several advancements in molecular genetics have allowed pathogenic mutations in diagnosing and classifying unique skin infections, such as psoriasis, atopic dermatitis, and skin cancer [124]. Advancements in molecular genetics also allowed the development of targeted and personalized therapeutics based on RNA interference [97]. Modification in RNA therapies utilized small RNA subunits in the form of small interfering RNAs (siRNA) for overexpressing the specific genes of the related disorders [98]. Therefore, Daniel Chin Shiuan Lio et al. developed mesoporous silica NPs nucleotide complexes of 200 nm with a pore size of 4 nm and mixed it with oligonucleotides of RNA, followed by overnight stirring. After stirring, the mesoporous silica NPs-oligonucleotide conjugate were coated with poly-L-lysine (PLL) in 1:1 for 10 min [108]. The excessive amount of PLL was removed via centrifugation at the speed of 10,000 rpm for 15 min. Excessive PLL-removed NPs were again re-suspended in the phosphate-buffered saline (PBS). Characterization of the PLL-coated nanocarriers was done via size analysis, labeling cells with MSNPs for the time-course study, and post-treatment in-vivo studies with a xenograft tumor model, primer sequence, real-time-polymerase chain reaction (RT-PCR), histological sectioning, Western blot, and flow cytometry. Results concluded that the loading of NPs-based oligonucleotides by poly-L-lysine resulted in improved transdermal drug delivery, increased zeta potential, and enhanced stability [125]. The MSNPs-PLL evaluation on skin squamous cell carcinoma (SCC) cells in-vitro showed a safety profile and increased penetration. Therefore, we are bound to believe that MSNPs-PLL proved to be accomplished candidates for non-invasive transdermal drug delivery in alleviating the skin cancer cells division [108].

Mesoporous silica NPs (MSNs) have been considered the most promising nanocarriers for attached targeted moieties owing to unique features of tunable pore structure, greater surface area, and pore volume [126,127,128]. These flexible features of the MSNs are accountable for successful conjugation, thermal stability, and biocompatibility. Colorectal cancer is a heterogeneous and lethal disease, proceeding towards the development of malignant tumors in the inner walls of the colon and rectum in the form of polyps. MicroRNA-155 (miR-155) is an oncogenic microRNA, and is over-expressed in many cancers, including colorectal cancer (CRC). Therefore, targeting the approach in treating cancers by using miR-155 is a nominal strategy for treating cancer. Therefore, Yang Li (2018) [104] developed anti-miR-155-loaded MSNs functionalized with polymeric dopamine (PDA) and AS1411 aptameric (MSNs-anti-miR-155@PDA-Apt) for the targeted therapy of CRC. The prepared NPs were characterized based on size, cell uptake studies, in-vitro cytotoxicity, xenograft tumor model, in-vivo imaging and biodistribution, in-vivo antitumor efficacy, and systemic toxicity. The results showed that miR-155 was highly over-expressed in CRC tissues, resulting in a significantly high targeted therapy and enhanced therapeutic efficacy [104].

## 4. Advantages and Limitations of RNA-Based Nano-Theranostic Systems

Thanks to their selectivity and enhanced sensitivity, RNA-based theragnostic approaches have tremendously improved current methods for diagnosis and provided versatile delivery systems for the treatment of a variety of cancers. This is important because in-vivo applications of the naked RNA molecule face many challenges that need to be tackled. For instance, the naked RNA molecules have poor chemical solubility, extremely short half-lives, and are easily degraded by nucleases [129,130,131]. Interestingly, developed nanovehicles protect the RNA molecule from immune system threats and degradation by nuclease enzymes and enhance the EPR effect, thus enabling the accumulation of the RNA molecule in the cancerous sites [130,132]. In terms of cancer therapy, however, there are benefits and drawbacks to applying such innovative modalities. RNA-nanovehicles functionalized with different moieties have enhanced cargo delivery without adverse side effects, and thus, can be considered efficient and targeted strategies for delivering chemotherapeutic agents to malignant cells.

A number of nanovehicles have been studied for their ability to deliver RNA molecules to target locations. For instance, it has been documented that lipid-based nanostructures (i.e., liposomes, solid lipid NPs, and lipid emulsions), polymer-based nanomaterials, inorganic NPs, and bio-inspired nanovehicles can be used for the targeted delivery of nucleic acids, such as RNA. The majority of these nanocarriers have the advantages of easy preparation, good biocompatibility and bioavailability, low cost of production, controlled release, easy modification, and easy uptake. However, they have shown poor solubility, rapid clearance, easy leakage of cargo, dose-dependent toxicity, and stability concerns [112].

The application of RNA technology has a major advantage of exploiting cellular machinery that permits the targeting of complementary transcripts, resulting in a dramatic decrease in the expression of a target gene. The main limitations of this approach are ineffective in-vivo delivery, off-target effects, and the induction of type I interferon responses. [129].

**Figure 7:**
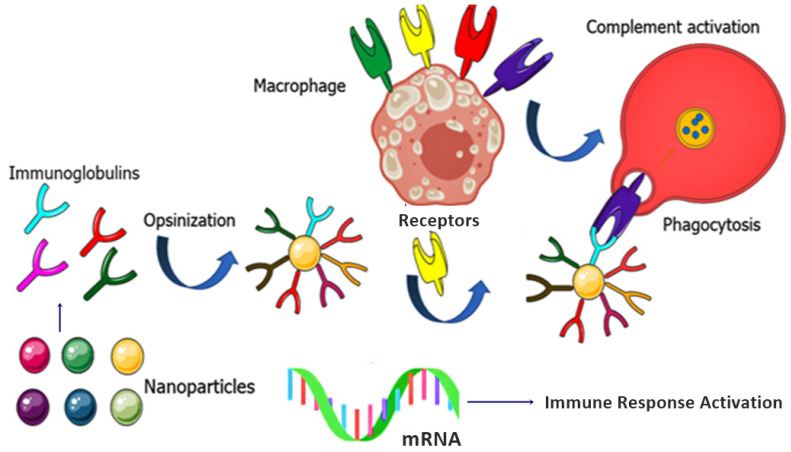
mRNA-based vaccines impart immunoglobulins and immune responses, leading towards phagocytosis.

The RNA molecule itself is unable to cross cell membranes. In addition, prompt degradation of the therapeutic agent in the endocytic pathway, called the endosomal escape, could be a great challenge in ligand-mediated endocytosis for the specific delivery of siRNA [133]. A crucial limitation for in-vivo delivery of siRNAs is the size of the synthesized RNA NPs. Basically, the average size of an RNA nanostructure is about 20 to 40 nm, which enhances its biodistribution in the blood circulation system, while the average size of a normal single siRNA molecule is less than 10 nm. Moreover, non-formulated siRNAs can be easily excreted through the urinary system [133,134,135]. Chemical and thermodynamic instability, short in-vivo half-life, undesirable in-vivo toxicity of RNA NPs along with targeting problems, high production costs, and a low yield are the primary drawbacks of the application of RNA nanostructures for theranostic purposes [133]. Using targeting moieties, such as aptamers or receptor-targeting ligands (i.e., folate), can improve RNA delivery. Still, endosomal escape might be a fundamental limitation that needs to be overcome shortly. Designing novel targeting RNA NPs with the ability to escape endosomes will lead the way toward more promising RNA therapeutics. mRNA-based vaccines impart immunoglobulins and immune responses, leading towards phagocytosis, as shown in Figure 7. Moreover, a list and links to information about clinical trials regarding RNA-based nanomaterials against cancer is now given in Table 3.

## 5. Conclusions and Future Prospective

In this review, we highlighted advancements in the use of RNA nanotechnology in cancer diagnosis and treatment. The development of novel RNA nanotechnology-based tools for cancer diagnosis and treatment has been studied extensively in recent years. Compared to presently available cancer diagnosis and treatment in clinics, a number of RNA-based NPs demonstrated improved sensitivity and selectivity or provided whole new capabilities that could not be attained with conventional techniques. Compared with conventional diagnosis and therapies, an RNA functionalized nanocarrier-mediated anticancer drug delivery leads to high therapeutic efficacy, targeted binding with the ligand, more accurate diagnosis, lower toxicity, and site-specific delivery, resulting in cytotoxicity management and cost-effectiveness. The barriers to the diagnosis and treatment of cancers and the killing of healthy cells have been minimized using biocompatible polymers ligands. Promising advances in cancer treatment and detection, such as RNA-functionalization and biocompatible ligands, are rapidly paved the way in addressing the disadvantages of existing approaches by effectively enhancing the treatment and diagnosis of metastatic tumors. Recently, significant advances have been made in the area of RNA nanotechnology for cancer diagnosis and treatment, and our knowledge of this topic has significantly expanded. However, just a few RNA-based NPs have progressed to clinical trials, and RNA nanotechnology is expected to enter the clinic in the coming years with collaborative efforts among scientists, technologists, and therapists. RNA nanotechnology, with its excellent specificity, accuracy, and multiplexed measuring capabilities, has significant potential for improving cancer diagnosis and treatment, finally leading to a higher cancer patients’ chance of survival.

## Figures and Tables

**Table 2:** Summary of RNA-based nanostructures in treatment of cancers.

Nanostructure	Key Feature	Ref
Ultra-thermostable RNA NPs	RNA ligand proved to dramatically inhibit the growth of breast cancer with non-detectable toxicity and immune responses in mice.	[102]
Selenium-siRNA NPs	Small interfering RNA (siRNA) showed great potential in advanced therapeutics because of its highly sequential ability for silencing HeLa genes for cervical cancer	[103]
MSN-anti-miR-155 NPs	miR-155 was highly over-expressed in colorectal tissues and cell lines as compared to the control groups and showed enhanced therapeutic efficacy.	[104]
Survivin-siRNA NPs	The novel nanocarrier system was able to initiate a specified and safe cellular uptake with increased transfection efficacy, promoting the downregulation of HCC cells.	[105]
Enveloped siRNA NPs	siRNA multi-functionalized nano-enveloped carriers can strongly silence target genes expressions as well as strongly pre-dominant genes, such as prohibitin 1 (PHB1), resulting in significantly culminating prostate tumor growth	[106]
FA-PEI-Fe_3_O_4_-siRNA NPs	Effective targeted PD-L1-knockdown therapy as well as a diagnosis in gastric cancers, thus favoring towards the best theranostic approach	[107]
PLL-siRNA-MSN NPs	MSNPs-PLL proved to be an accomplished candidate for non-invasive transdermal drug delivery in alleviating skin cancer cells division	[108]

**Table 3:** List and links to information about clinical trials regarding RNA-based nanomaterials against cancer.

RNA Based Nanomaterials	Clinical Trials	Ref.
The self-delivering RNA (sd RNA)	Combination of immunotherapy and chemotherapy for cancer treatment in pre-clinical trials.	[136]
Single mRNA-4157vaccine	Preclinical phase 2 against melanoma.	[137]
Adjuvant claudin mRNA cells	Pre-clinical stages against metastatic breast cancer.	[138]
mRNA 5671 based NPs	Pre-clinical stages against colorectal cancer, lungs cancer, and pancreatic cancer.	[139]
mRNA 2416 based NPs	Pre-clinical stages against solid tumors in ovarian cancer.	[140]

## Data Availability

Data sharing is not applicable to this article as no new data were created or analyzed in this study.

## Abbreviations

NPs	Nanoparticles
siRNAs	Short interfering RNAs
PTX	Paclitaxel
SPR	Surface plasmon resonance
PRRs	Pattern recognition receptors
EPR	Enhanced permeability and retention
AuNPs	Gold nanoparticles
QDs	Quantum dots
ssDNA	Single-stranded DNA
SELEX	Systemic Evolution of Ligands by EXponential enrichment
NIRF	Near-infrared fluorescent
MBS	m-maleimidobenzoyl N-hydroxysuccinimde ester
GBM	Glioblastoma multiforme
iMER	Immune-Magnetic Exosome RNA
EGFR	Epidermal growth factor receptor
GSCs	GBM stem cells
TICc	Tumor-initiating cells
TNBC	Triple-negative breast cancer
CTCs	Circulating tumor cells
PSA	Prostate-specific antigen
CLs	Cationic lipids
PEG	Polyethylene glycol
ECM	Extracellular matrix
PD-L1	Programmed death-ligand 1
TEM	Transmission electron microscopy
FTIR	Fourier transform infrared spectroscopy
EDS	Energy dispersive spectroscopy
ROS	Reactive oxygen species
PHB1	Prohibitin 1
FA	Folic acid
MRI	Magnetic resonance imaging
HCC	Hepatocellular carcinoma
CRC	Colorectal cancer
PLL	Poly-L-lysine
PBS	Phosphate-buffered saline
RT-PCR	Real-time-polymerase chain reaction
MSNs	Mesoporous silica nanoparticles
PDA	Polymeric dopamine
